# Treatment of advanced medullary thyroid cancer with an alternating combination of doxorubicin-streptozocin and 5 FU-dacarbazine

**DOI:** 10.1054/bjoc.2000.1314

**Published:** 2000-08-17

**Authors:** M Nocera, E Baudin, G Pellegriti, A F Cailleux, C Mechelany-Corone, M Schlumberger, the Groupe d'Etude des Tumeurs a Calcitonine (GETC)

**Affiliations:** Service de Médecine Nucléaire et de Cancérologie Endocrinienne, Institute Gustave-Roussy, Villejuif Cedex, 94805, France; Service de Médecine Nucléaire, Centre René Huguenin, Saint-Cloud, 92210, France

**Keywords:** chemotherapy, medullary thyroid carcinoma

## Abstract

Combinations of doxorubicin and streptozocin and 5-FU and dacarbazine were given alternately to 20 patients with metastatic medullary thyroid carcinoma. Three partial responses and 10 long-term stabilizations were observed. No unexpected toxicity occurred. © 2000 Cancer Research Campaign


*British Journal of Cancer* (2000) **83**(6), 715–718
© 2000 Cancer Research Campaign
doi: 10.1054/ bjoc.2000.1314, available online at http://www.idealibrary.com on IDEAL®

### Treatment of advanced medullary thyroid cancer with an alternating combination of doxorubicin-streptozocin and 5 FU-dacarbazine

**M Nocera[1], E Baudin[1], G Pellegriti[1], AF Cailleux[1], C Mechelany-Corone[2], M Schlumberger[1] and the Groupe d'Etude des Tumeurs à Calcitonine (GETC)**

[1]Service de Médecine Nucléaire et de Cancérologie Endocrinienne, Institute Gustave-Roussy, 94805 Villejuif Cedex, France; [2]Service de Médecine Nucléaire, Centre René Huguenin, 92210 Saint-Cloud, France

**Summary** Combinations of doxorubicin and streptozocin and 5-FU and dacarbazine were given alternately to 20 patients with metastatic medullary thyroid carcinoma. Three partial responses and 10 long-term stabilizations were observed. No unexpected toxicity occurred. © 2000 Cancer Research Campaign

**Keywords:** chemotherapy; medullary thyroid carcinoma

Medullary thyroid carcinoma (MTC) is a neuroendocrine tumour which arises from thyroid C-cells. It accounts for 5 to 10% of all thyroid cancers. Total thyroidectomy with lymph node dissection is the main treatment when the disease is confined to the neck (Chi and Moley, 1998; Schlumberger and Pacini, 1999). External irradiation to the neck may be indicated in the absence of known distant disease, in patients with a detectable CT level after an apparently complete surgical procedure, and in those with an inoperable disease (Tubiana et al, 1985; Brierley et al, 1996; Fife et al, 1996). This procedure reduces the recurrence rate in the neck, but its impact on patients' survival has still to be demonstrated. Chemotherapy plays no role in the early management (Chi and Moley, 1998; Schlumberger and Pacini, 1999).

This disease usually follows an indolent course even at the stage of distant spread, and patients with lung or liver metastases may survive years without systemic treatment (Saad et al, 1984; Tubiana et al, 1985; Van Heerden et al, 1990; Chi and Moley, 1998; Brierley et al, 1996). In a minority of these metastatic patients, chemotherapy may be indicated for rapidly progressive distant metastases (Cailleux and Schlumberger, 1998).

Chemotherapy trials have been limited by the scarcity of these tumours. Furthermore, due to the indolent course of the disease and the poor results of chemotherapy, only patients with large tumour burden or rapidly progressing distant metastases have been included in these trials. Response rates were low with doxorubicin (Shimaoka et al, 1985; Droz et al, 1990) and cisplatin (Hoskin and Harmer 1987; Droz et al, 1990), given either as single agents or in combination (Shimaoka et al, 1985; Williams et al, 1986; De Besi et al, 1991). Furthermore, toxicities were significant.

*Received 18 January 2000*
*Revised 26 April 2000*
*Accepted 1 May 2000*

*Correspondence to:* M Schlumberger

A trial (Schlumberger et al, 1995) with etoposide given as a single agent did not confirm a previous report (Hoskin and Harmer, 1987) as no tumour response was observed among 16 evaluable patients.

Combination of drugs which have been shown to be active in the treatment of other neuroendocrine tumours may provide further therapeutic options in MTC patients. Indeed, several trials carried out on limited numbers of MTC patients have shown that these drugs are active in some. With the combination of 5FU-dacarbazine, a complete response was observed in one patient (Pertusson, 1988), and 3 partial responses were observed among 5 patients (Orlandi et al, 1994). Two partial responses were observed among 7 patients treated with cyclophosphamide, vincristine and dacarbazine (Wu et al, 1994). With a combination of doxorubicin and streptozocin, one partial response was obtained among 5 patients (Frame et al, 1988), and using a combination of epirubicin, 5-FU and dacarbazine, one partial response was observed among 7 patients (Di Bartolomeo et al, 1995). In a previous study (Schlumberger et al, 1995), combinations of 5-FU/streptozocin and 5-FU/dacarbazine were given alternatively to 20 patients. Three partial responses and 11 stabilizations were observed.

Considering these results, the alternating combinations of doxorubicin/streptozocin and 5-FU/dacarbazine, that have already been used in gastro-entero-pancreatic neuroendocrine tumours (Moertel et al, 1994), were given to 20 patients with progressive metastatic MTC.

### PATIENTS AND METHODS

### Patients

From October 1994 to February 1999, 20 patients (Table 1) with progressive distant metastases of MTC were entered into this trial. Their mean age was 49 years (range 30–79). There were 14 males and 6 females. Among them, 16 had undergone a total thyroidectomy with bilateral cervical lymph node dissection, and 10 had

**Table 1** Clinical characteristics of the twenty patients included in the trial

| Patient | Sex/Age (years) | Type | Metastatic sites | Surg | RT | PS | No of courses | Tumour response | Marker response CT | Marker response CEA | Toxicity (WHO grade) | Response duration (months) | Survival after chemotherapy (months) |
|---|---|---|---|---|---|---|---|---|---|---|---|---|---|
| 1 | M/52 | S | Lungs, bones, liver | + | – | 0 | 6 | PR | PR | NC | Vomiting (2) | 27 | 33D |
| 2 | F/63 | S | Nodes, trachea | + | – | 0 | 4 | NC | PR | P | Vomiting (3) | 16 | 31D |
| 3 | M/45 | S | Lungs, bones, liver nodes | + | + | 0 | 6 | PR | PR | PR | Cardio (3) | 18 | 39A |
| 4 | M/46 | S | Lungs, bones nodes | + | + | 2 | 3 | P | NC | NC | Diarrhea(3) | – | 12D |
| 5 | M/58 | S | Liver | + | + | 0 | 6 | NC | NC | NC | Cardio (3) | 18 | 24D |
| 6 | M/69 | S | Lungs, bones nodes | + | – | 0 | 6 | NC | NC | PR | Leucopenia (4) | 13+ | 13A |
| 7 | M/69 | S | Lungs, bones, liver | + | – | 3 | 2 | P | P | P | – | – | 12A |
| 8 | M/38 | F | Lungs, liver | + | – | 0 | 4 | NC | NC | NC | – | 8+ | 8A |
| 9 | M/30 | S | Lungs, bones | + | – | 0 | 6 | NC | NC | NC | Liver(2) | 12+ | 12A |
| 10 | M/43 | S | Lungs, bones, liver nodes, spleen | – | – | 1 | 2 | P | P | NC | – | – | 19D |
| 11 | M/50 | S | Trachea | + | + | 0 | 4 | PR | PR | – | – | 28+ | 28A |
| 12 | F/42 | F | Bones, liver Lungs, nodes | + | + | 0 | 6 | NC | P | P | Stomatitis (2) | 48 | 60A |
| 13 | F/47 | S | Bones, liver Lungs, nodes | + | + | 0 | 6 | NC | P | NC | – | 14 | 19A |
| 14 | F/79 | S | Bones, liver | + | + | 1 | 2 | P | P | P | – | – | 8D |
| 15 | M/40 | S | Bones, liver Lungs, nodes | + | – | 0 | 6 | NC | P | NC | Alopecia (4) | 51+ | 51A |
| 16 | F/39 | S | Lungs, liver | + | + | 0 | 4 | NC | PR | PR | Vomiting (2) | 25 | 39A |
| 17 | F/34 | S | Lungs, nodes Pleura | + | + | 0 | 5 | NC | PR | P | Alopecia (3) | 25 | 34A |
| 18 | M/60 | S | Lungs, nodes | – | + | 0 | 4 | P | P | P | Leucopenia (3) | – | 18D |
| 19 | M/32 | S | Lungs, liver | – | – | 1 | 2 | P | P | P | Vomiting (2) | – | 24A |
| 20 | M/51 | S | Bones, liver Lungs, nodes | – | – | 0 | 3 | P | P | P | – | – | 26A |

M: males; F: females; S: sporadic; F: familial; RT: cervico-mediastinal irradiation; PS: performance status; CT: calcitonin; CEA: carcinoembryonic antigen; PR: partial response; NC: no change; P: progression; D: death; A: alive received post-operatively external radiotherapy to the neck and mediastinum. Four patients were not operated on for diffuse distant metastases at presentation or locally advanced disease. None of them had previously been treated with chemotherapy. Median follow-up since the diagnosis was 4.5 years (range, 2.3–37 years).

All patients had measurable metastatic lesions that were documented by chest radiography, neck or liver ultrasonography, and neck, chest or abdominal CT scan or magnetic resonance imaging. These lesions have progressed during the preceding 3–6 months. The tumour markers calcitonin (CT) and carcinoembryonic antigen (CEA) were measured and followed in all patients but were not accepted as the only response criteria.

Other eligibility requirements were as follows: ECOG status <2, no major ECG changes and an ejection fraction >50% as measured by an isotopic technique; leucocyte count greater than 4000 $\mu l^{-1}$ and platelet count greater than 150 000 $\mu l^{-1}$, a serum creatinine of 120 $\mu mol\ l^{-1}$ or less, a 24 h proteinuria <200 mg, a total bilirubin of less than or equal to 34 $\mu mol\ l^{-1}$.

All patients were evaluated upon entry into the study, and after every 2 courses of chemotherapy, with clinical staging, CT and CEA measurements and morphological investigations including chest X-ray, neck and liver ultrasonography and chest or abdominal CT scan. Routine chemistry and ECG were performed before each course of chemotherapy.

The study was approved by the local Ethics Committee and an informed consent was obtained from all patients.

### Treatment

The chemotherapy protocol consisted in doxorubicin 60 mg/m$^2$ i.v. in 5 minutes on day 1, and streptozocin 500 mg/m$^2$ in 4 hours perfusion, daily for 5 consecutive days. Four weeks later, 5-FU was given at 400 mg/m$^2$, and dacarbazine at 200 mg/m$^2$, both by intravenous injection daily for 5 consecutive days. At 8 weeks, this cycle was repeated.

Three categories of response were assessed: (1) tumour, (2) biochemical, and (3) symptomatic. The criteria used for reporting responses for measurable tumour masses were those of Miller et al (1981): partial remission (PR) was defined as greater than or equal to 50% decrease in the sum of the products of the two largest perpendicular diameters of all tumour masses for at least 1 month; no change (NC), defined as less than 50% decrease or less than 25% increase in the size of measurable lesions; progressive

 *© 2000 Cancer Research Campaign*

disease (P) defined as greater than or equal to 25% increase in any tumour lesion or the appearance of new site. For biochemical response, PR was defined as greater than or equal to 50% decrease for at least 1 month. For symptomatic response, PR was defined as a reduction of at least 50% in both the frequency and intensity of flushing and/or diarrhoea attacks. In those patients who achieved a partial response, the period of overall duration of response was calculated from the first day of treatment to the date of first observation of progressive disease.

Toxicity was evaluated monthly and complete re-staging was performed after 2, 4 and 6 cycles of chemotherapy. Therapy was continued until tumour progression, and in cases of tumour response or stabilization for a total of 6 cycles (12 months), and as long as there was no evidence of symptomatic or general deterioration or toxicity.

### RESULTS

The 20 patients entered into this trial were evaluable for toxicity and response. Each patient received an average of 4 (range 2 to 6) cycles of doxorubicin-streptozocin and 5-FU-dacarbazine.

Four of the 5 patients with invalidating symptoms before starting chemotherapy had a partial symptomatic response.

Three partial tumour responses were obtained after 3, 2 and 3 cycles of chemotherapy, and lasted 27, 28+ and 18 months, respectively. Calcitonin level decreased by 65%, 73% and 58% respectively, and CEA level was stable in one patient, was normal in one before therapy, and decreased by 60% in one.

Ten patients had a stable disease for 8–51 months (mean 23 months). Among these 10 patients, 3 had progressive marker levels, 4 had stable marker levels and 3 had a partial biological response. Seven patients had progressive disease.

Nausea and vomiting were limited by antiemetic drugs and digestive toxicity was observed in 5 patients (grade 2 in 3 and grade 3 in 2). One patient had a grade 2 stomatitis. Hepatic toxicity (grade 2) was observed in one patient, haematologic toxicity in 2 patients (grade 3 and 4), and cardiac toxicity in two patients (grade 3). There were no treatment-related deaths.

With a mean follow-up of 25 months (range, 8–60 months) since the beginning of therapy, seven deaths occurred, including one among the 3 patients who had responded to therapy, 2 among the 10 patients who achieved a stabilisation of the disease, and 4 in the 7 patients with progressive disease.

### DISCUSSION

The alternating combination of doxorubicin-streptozocin, and 5-FU-dacarbazine, was used because it combines drugs that have been shown to be active in medullary thyroid carcinoma, when used separately; also, this association has been shown to be active in other neuroendocrine tumours, with an acceptable toxicity (Moertel et al, 1994).

In 20 patients with metastatic MTC, it resulted in 3 partial responses (15%), and in 10 long-term stabilisations (50%). Considering that all patients included in the trial were affected by a rapidly progressing disease, these results confirm the potential benefits of cytotoxic chemotherapy, and are in agreement with the results obtained with the association of epirubicin, dacarbazine and 5-FU (Di Bartolomeo et al, 1995).

Nevertheless, the response rate was not improved by adding doxorubicin to the previously tested regimen of streptozocin plus 5-FU and dacarbazine, and toxicity was slightly higher (Schlumberger et al, 1995). Therefore, we consider the previous regimen as the reference combination for metastatic MTC patients with rapidly progressing disease. The absence of synergistic effect among these cytotoxic agents can be attributed to the large size of the neoplastic masses and also to intrinsic cellular factors determining chemoresistance (Yang et al, 1991).

Nevertheless, because of the high metastatic potential of medullary thyroid carcinoma, further studies are necessary in order to identify new therapeutic strategies with cytotoxic or cytostatic molecules that will permit the treatment of these patients at an earlier stage.

### REFERENCES

Brierley J, Tsang R, Simpson WJ, Gospodarowicz M, Sutcliffe S and Panzarella T (1996) Medullary thyroid cancer: analysis of survival and prognostic factors and the role of radiation therapy in local control. *Thyroid* **6**: 305–310

Cailleux AF and Schlumberger MJ (1998) Chemotherapy and biological therapy of thyroid cancer. In: *Thyroid cancer* J Fagin (ed), Kluwer Academic Publishers, Boston. pp. 341–350

Chi DD and Moley JF (1998) Medullary thyroid carcinoma: genetic advances, treatment recommendation and the approach to the patient with persistent hypercalcitoninemia. *Surg Oncol Clin N Am* **7**: 681–706

De Besi P, Busnardo B, Toso S, Girelli ME, Nacamulli D, Simioni N, Casara D, Zorat P and Fiorentino MV (1991) Combined chemotherapy with bleomycin, adriamycin and platinum in advanced thyroid cancer. *J Endocrinol Invest* **14**: 475–480

Di Bartolomeo M, Bajetta E, Bochicchio AM, Carnaghi C, Somma L, Mazzaferro V, Visini M, Gebbia V, Tumolo S and Ballatore P (1995) A phase II trial of dacarbazine, fluorouracil and epirubicin in patient with neuroendocrine tumors. A study by the italian trials in medical oncology (ITMO) group. *Ann Oncol* **6**: 77–79

Droz JP, Schlumberger M, Rougier P, Ghosn M, Gardet P and Parmentier C (1990) Chemotherapy in metastatic non anaplastic thyroid cancer: experience at the Institut Gustave-Roussy. *Tumori* **76**: 480–483

Fife KM, Bower M and Harmer CL (1996) Medullary thyroid cancer: the role of radiotherapy in local control. *Eur J Surg Oncol* **22**: 588–591

Frame J, Kelsen D, Kemeny N, Cheng E, Niedzwiecki D, Heelan R and Lipperman RA (1988) A phase II trial of streptozotocin and adriamycin in advanced APUD tumors. *Am J Clin Oncol* **11**: 490–495

Hoskin PJ and Harmer C (1987) Chemotherapy for thyroid cancer. *Radiother Oncol* **10**: 187–194

Miller AB, Hoogstraten B, Staquet M and Winkler A (1981) Reporting results of cancer treatment. *Cancer* **47**: 207–214

Moertel CG, Johnson CM, McKusick JK Jr, Nagorney DM, Kvols LK, Rubin J and Kunselman S (1994) The management of patients with advanced carcinoid tumors and islet cell carcinomas. *Ann Intern Med* **120**: 302–309

Orlanci F, Caraci P, Berruti A, Puligheddu B, Pivano G, Dogliotti L and Angeli A (1994) Chemotherapy with dacarbazine and 5-fluoro-uracil in advanced medullary thyroid cancer. *Ann Oncol* **5**: 763–765

Petursson SR (1988) Metastatic medullary thyroid carcinoma: complete response to combination chemotherapy with dacarbazine and 5-fluoro-uracil. *Cancer* **62**: 1899–1903

Saad MF, Ordonez NG, Rashid RK, Guido JJ, Hill CS, Hickey RC and Saaman NA (1984) Medullary carcinoma of the thyroid. A study of the clinical features and prognostic factors in 161 patients. *Medicine (Baltimore)* **63**: 319–342

Schlumberger M and Pacini F (1999) Medullary thyroid carcinoma. In: *Thyroid tumors*. Editions Nucléon, Paris. pp 267–299

Schlumberger M, Abdelmoumene N, Delisle MJ and Couette JE (1995) Treatment of advanced medullary thyroid cancer with an alternating combination of 5 FU-streptozocin and 5 FU-dacarbazine. *Br J Cancer* **71**: 363–365 365.

Shimaoka K, Schoenfeld DA, De Wys WD, Creech RH and De Conti R (1985) A randomized trial of doxorubicin versus doxorubicin plus cisplatin in patients with advanced thyroid carcinoma. *Cancer* **56**: 2155–2160

© 2000 Cancer Research Campaign 

Tubiana M, Haddad E, Schlumberger M, Hill C, Rougier P and Sarrazin D (1985) External radiotherapy in thyroid cancers. *Cancer* **55**: 2062–2071

Van Heerden JA, Grant CS, Gharib H, Hay ID and Illstrup DM (1990) Long-term course of patients with persistent hypercalcitoninemia after apparent curative primary surgery for medullary thyroid carcinoma. *Ann Surg* **212**: 395–401

Williams SD, Birch R and Einhorn LH (1986) Phase II evaluation of doxorubicin plus cisplatin in advanced thyroid cancer: a southeastern cancer study group trial. *Cancer Treat Rep* **70**: 405–407

Wu LT, Averbuch SD, Ball DW, De Bustros A, Baylin SB and Mac Guire III WP (1994) Treatment of advanced medullary thyroid carcinoma with a combination of cyclophosphamide, vincristine and dacarbazine. *Cancer* **73**: 432–436

Yang KP, Liamg YF and Samaan NA (1991) Intrinsic drug resistance in a human medullary thyroid carcinoma cell line: association with overexpression of mdrl gene and low proliferation fraction. *Anticancer Res* **11**: 1065–1068

*© 2000 Cancer Research Campaign*
